# Network recovery based on system crash early warning in a cascading failure model

**DOI:** 10.1038/s41598-018-25591-6

**Published:** 2018-05-10

**Authors:** Dong Zhou, Ahmed Elmokashfi

**Affiliations:** Simula Metropolitan CDE, Fornebu, 1364 Norway

## Abstract

This paper investigates the possibility of saving a network that is predicted to have a cascading failure that will eventually lead to a total collapse. We model cascading failures using the recently proposed KQ model. Then predict an impending total collapse by monitoring critical slowing down indicators and subsequently attempt to prevent the total collapse of the network by adding new nodes. To this end, we systematically evaluate five node addition rules, the effect of intervention delay and network degree heterogeneity. Surprisingly, unlike for random homogeneous networks, we find that a delayed intervention is preferred for saving scale free networks. We also find that for homogeneous networks, the best strategy is to wire newly added nodes to existing nodes in a uniformly random manner. For heterogeneous networks, however, a random selection of nodes based on their degree mostly outperforms a uniform random selection. These results provide new insights into restoring networks by adding nodes after observing early warnings of an impending complete breakdown.

## Introduction

Cascading failures and the recovery from them is one of the most popular research directions in network science. Recently, the percolation theory has been widely used for modeling cascading failures in interdependent networks, where failures propagate among networks due to predefined dependency links^1–9^. Overload-triggered cascades in single or coupled networks have also been the subject of much work in the past decade^10–21^. Besides the above mentioned models, other models like *k*-core cascades, sandpile models have also been employed for understanding failure propagation and systems collapse^22–27^. Based on the above modeling frameworks of cascading failures, different approaches for system repair have also been studied. Most of these works consider including rules for restoring nodes that fail during the cascading failure process^28–33^. For example, A. Majdandzic, *et al*. in 2014 presented a model, where a node recovers from an internal or external failure after a fixed period of time. This model leads to an interesting phase-flipping phenomena, as well as a strong hysteresis behavior^28^. This model was later extended by using a randomized recovery method^31^. More recently, M.A. Di Muro, *et al*. studied a node repairing strategy for interdependent random networks, where a failed node can be repaired with a certain probability if it is a part of the current giant connected components^32^. A. Majdandzic, *et al*. further studied the cascade and node recovery model for multi-layer interacting networks and also investigated the optimal repairing strategy for a collapsed coupled system^33^.

Many cascading failure models exhibit the interesting phenomena of “critical slowing down”: systems near criticality can experience a much longer cascading process (the so called “plateau stage”), which is sensitive to noise, before a final total collapse^1,34–36^. For example, D. Zhou, *et al*. studied the branching process behind the critical cascading failures in interdependent networks, and showed the critical/non-critical scaling rules of the total cascade length^34^. In addition, G.J. Baxter, *et al*. studied the critical and non-critical dynamic processes in the *k*-core pruning model^35^. Recently, D. Lee, *et al*. presented a universal model for hybrid percolation transitions and investigated the resulting critical cascading process^36^. Most of these studies mainly focused on interpreting the time length of the critical slowing down phase. Further, early warning indicators for system transitions based on the critical slowing down have already been evaluated for many real systems^37–42^. This technique has also been used for predicting system collapse in cascading failure models. For example, B. Podobnik, *et al*. studied indicators to predict total collapses in a cascading failure model on random networks^43^.

Although the critical slowing down phenomena have been leveraged to provide indicators of impending cascades, there is still an important open question: how to restore the system after an early warning has been recorded? In this work, we attempt to investigate and answer this question. To this end, we have systematically explored several system recovery strategies after observing an early warning of a total system crash. We base our work on the recently proposed model of cascading failures by Y. Yu, *et al*.^44^. This cascading failure model is an extension of the *k*-core cascade, where a node will be removed from the network with a probability *f* if it has fewer than *k*_*s*_ connections, or it has lost more than a fraction 1 − *q* of its original neighbors. Further, as in^43^, we employ the moving standard deviation (MVSD) of the remaining system size time series as an early indicator of an impending cascade. We then compare five different node-addition based recovery strategies and study the effect of response time delay on system recovery. We find that, for homogeneous Erdös-Rényi (ER) networks, an earlier node addition leads to a larger survival ratio. However, for scale-free (SF) networks, a delayed recovery can be better in some cases. We also find, for ER networks, that it is always better to connect the newly added nodes to existing nodes in a uniformly random manner. However, for SF networks, a roulette selection based on each node’s original degree (or its reciprocal) can perform better for earlier node additions. These results provide insights on how to save a system that has been predicted to collapse.

## Results

### Cascading failure model and recovery strategies

**Figure 1:**
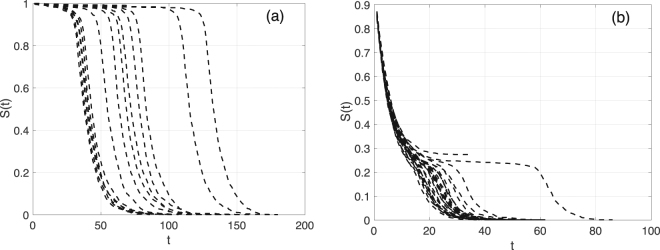
Examples of the cascading failure process near criticality. (**a**) 30 examples of the cascading failure process for ER networks with *N* = 1000, 〈*k*〉 = 20, *k*_*s*_ = 11, *q* = 0.09, and *f* = 0.1. (**b**) Similar to (a) but for SF networks with *γ* = 1.8, *k*_*s*_ = 5, *q* = 0.39, and *f* = 0.2.

In this work, we follow the KQ modeling framework of system crash introduced by Y. Yu, *et al*.^44^. A node will be removed from the system with a probability *f*, if it’s current degree is smaller than a threshold *k*_*s*_ or it has lost more than a fraction *q* of its original neighbors. The fraction of remaining nodes is used as a measure of the system robustness. The KQ model exhibits an interesting behavior for certain parameter values, where systems would experience a slow cascading failure process in a plateau stage (pseudo-steady states) before an abrupt total collapse. In the following, we focus on cases with sudden total collapse after a pseudo-steady state. Our goal is to investigate early warning indicators and compare system recovery strategies. We focus on two cases: ER networks with 〈*k*〉 = 20, *k*_*s*_ = 11, *q* = 0.09 and *f* = 0.1, and SF networks with *γ* = 1.8, *k*_*s*_ = 5, *q* = 0.39 and *f* = 0.2. These parameter values are inspired by the values used by Y. Yu, *et al*., who in turn based their choice of parameter values on measurements from real-world systems. We show 30 realizations of the cascading failure process for both ER and SF networks in Fig. 1. *S*(*t*), *t* = 1, 2, … denotes the proportion of remaining nodes at time step *t*. For both cases, the system is near criticality and has a plateau stage (pseudo-steady state) before reaching the final state (a total collapse or surviving near the plateau). Comparing Fig. 1(a) and Fig. 1(b), we find that the ER case has a plateau stage at around $S(t)\sim 1$, while the SF case has a plateau at around $S(t)\sim 0.2$. The latter has a much lower plateau stage, since the heterogeneous degree distribution leads to more failures at the beginning compared to ER networks. For ER networks, as long as the mean degree is significantly larger than the threshold *k*_*s*_, the system will have very few failures at the early time steps. In other words, the system size does not significantly change, which results in the observed plateau stage around 1.

In order to provide early warning indicators of a total collapse during the plateau stage, we need to capture both the beginning and the end of the plateau stage. To do this, we first define the moving standard deviation (MVSD) of *S*(*t*), MVSD(*t*), as the standard deviation (SD) of *S*(*t*) in time windows with length 5: *S*(*t* − 4), *S*(*t* − 3), …, *S*(*t*). For *t* ≤ 4, the first *t* values of *S*(*t*) will be used to calculate the MVSD instead. Note that the time series after the current time step *t* is not used, since we aim to provide early warning prediction based on historical records. We use a window length of 5 for calculating the MVSD, because some realizations for the SF network, as shown in Fig. 1(b), can reach a total collapse within 20 time steps.

We define the beginning of the plateau stage as *T*_start_ = 1 for the ER network case. For the SF network case, *T*_start_ is defined as the time step where MVSD(*t*) becomes smaller than 0.01 for the first time. This threshold is motivated by the observation that the MVSD will become smaller than 0.01 during the plateau stage in most cases. The end of the plateau stage, *T*_pred_, is defined as the first time step where MVSD(*T*_pred_) > mean(MVSD(*t* = *T*_start_, …, *T*_pred_ − 1)) + 3 · SD(MVSD(*t* = *T*_start_, …, *T*_pred_ − 1)). This definition is inspired by the fact that systems tend to have a continuously increasing SD when leaving the pseudo-steady state.

Following the prediction of the start and end of the plateau stage, we try to restore the system by adding *N*_*a*_ new nodes at time step *t* = *T*_pred_ + *T*_delay_, where *T*_delay_ ≥ 1 defines the time delay of the node addition process. Each of the additional nodes has *k*_*a*_ connections to *k*_*a*_ remaining nodes–If there are fewer than *k*_*a*_ remaining nodes, all of them will be connected to each additional node. Next we discuss different strategies for wiring the newly added nodes.

**“Uniformly random selection”:** at time step *T*_pred_ + *T*_delay_, each additional node is connected to *k*_*a*_ uniformly randomly sampled remaining nodes.

**“Largest degree selection”:** at time step *T*_pred_ + *T*_delay_, each additional node is connected to *k*_*a*_ remaining nodes that had the largest degree values in the original network.

**“Smallest degree selection”:** at time step *T*_pred_ + *T*_delay_, each additional node is connected to *k*_*a*_ remaining nodes that had the smallest degree values in the original network.

**“Roulette selection”:** at time step *T*_pred_ + *T*_delay_, each additional node is connected to *k*_*a*_ randomly selected remaining nodes, and the probability that a remaining node is selected is proportional to its degree in the original network.

**“Anti-roulette selection”:** at time step *T*_pred_ + *T*_delay_, each additional node is connected to *k*_*a*_ randomly selected remaining nodes, and the probability that a remaining node is selected is proportional to the reciprocal of its original degree.

**Figure 2:**
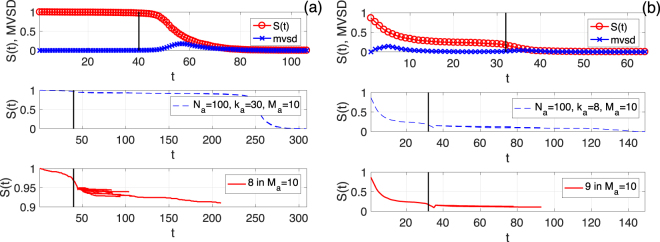
Examples of the system recovery after the early warning indicator. (**a**) One example of system recovery for ER networks with *N* = 1000 and 〈*k*〉 = 20. The uniformly random selection rule is used. *M*_*a*_ = 10, *k*_*s*_ = 11, *q* = 0.09, *f* = 0.1, *N*_*a*_ = 100, and *k*_*a*_ = 30. The threshold for determining a total collapse is *d* = 0.5. The upper panel shows the variation of *S*(*t*) (red line with circles) as well as the corresponding moving SD series (blue line with crosses). The black vertical line indicates the location of *T*_pred_. The middle panel shows all the 10 new time series (blue dashed lines) of *S*(*t*) after the node addition with *T*_delay_ = 6. The lower panel shows the 8 trials of node addition (red lines) without total collapses among the *M*_*a*_ = 10 trials in total. The threshold for determining a total collapse is *d* = 0.5. (**b**) Similar to (a) but for SF networks with *γ* = 1.8, *k*_*s*_ = 5, *q* = 0.39, *f* = 0.2, *N*_*a*_ = 100, *k*_*a*_ = 8 and *T*_delay_ = 5. For this example, there are 9 trials of node addition without total collapses within the 10 trials. The threshold for determining a total collapse is *d* = 0.1.

We use a threshold *d* for the fraction of remaining nodes, *S*(*t*), to determine if one realization of cascading failures in simulation has a total collapse. *d* is set to 0.5 and 0.1 for the ER and SF networks, respectively. These thresholds correspond to half the system sizes at the pseudo-steady states. For each realization with a total collapse, we repeat the node addition independently *M*_*a*_ times, and calculate a survival ratio over these *M*_*a*_ tests, *η*, which is the number of trials without total collapses divided by *M*_*a*_. We also find the time step, *t* = *T*_*d*_, where *S*(*t*) decreases to below the threshold *d*, after each trial of node addition with a total collapse. We repeat the above process for *M* realizations. To illustrate the above mentioned processes of total collapse prediction and mitigation via adding new nodes, we show in Fig. 2 examples for an ER network and a SF network. For both examples, we use *N*_*a*_ = 100 and *M*_*a*_ = 10. We, however, use different *k*_*a*_, *T*_delay_ values depending on the network: *k*_*a*_ = 30, *T*_delay_ = 6 for the ER network; and *k*_*a*_ = 8, *T*_delay_ = 5 for the SF network. These parameter values were carefully chosen to ensure that we do not end up with the extreme survival ratio *η* of 0 or 1. For node addition, we follow the uniformly random selection rule. The ER network survived in 8 out of the 10 trials, while 9 of them survived in the SF case (see the middle and lower panels of Fig. 2(a,b)). Therefore, the survival ratios for these two examples are 0.8 and 0.9, respectively.

### Comparisons of node addition rules

In the following, we investigate how different node addition rules impact the ability to recover a system with an impending total collapse for both ER and SF networks. We also study the role of the time delay *T*_delay_.

**Figure 3:**
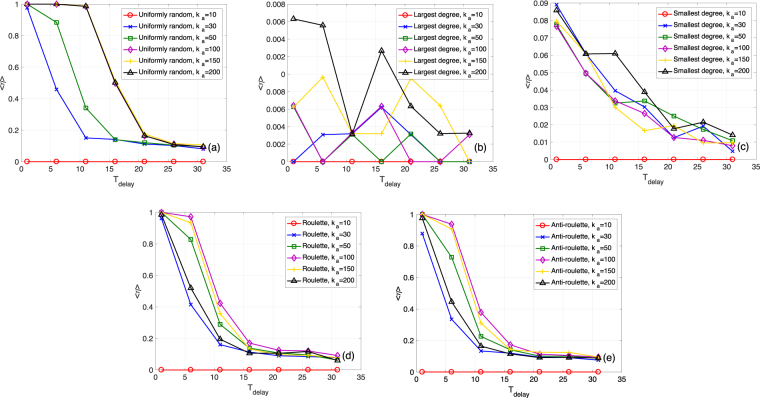
Mean survival ratio 〈*η*〉 as a function of *T*_delay_ for *N*_*a*_ = 100 and different *k*_*a*_ values. (**a**) ER networks with the uniformly random selection. *N* = 1000, *M* = 1000, *M*_*a*_ = 10, 〈*k*〉 = 20, *k*_*s*_ = 11, *q* = 0.09, and *f* = 0.1. The threshold for determining a total collapse is *d* = 0.5. (**b–e**) Similar to (a) but for the largest degree, smallest degree, roulette, and anti-roulette selection rules.

First, we focus on the ER network case with *N*_*a*_ = 100 and different values of *k*_*a*_. Fig. 3(a–e) show how the mean survival ratio 〈*η*〉 varies for different values of *k*_*a*_ as we vary *T*_delay_, for the five different approaches of node addition. For example, according to Fig. 3(a,d,e), for the three randomized selection rules, the survival ratio decreases from 1 to around 0 as *T*_delay_ increases or as *k*_*a*_ decreases. However, Fig. 3(b,c) show that, for the largest degree and smallest degree selection rules, the system has much lower survival ratios. This is because all the *N*_*a*_ ⋅ *k*_*a*_ additional links are added between *N*_*a*_ new nodes and the *k*_*a*_ remaining nodes with the largest or smallest original degrees. This will lead to a final state, with around *N*_*a*_ + *k*_*a*_ nodes, smaller than the threshold *d* = 0.5. We also notice that for the roulette/anti-roulette selection, when *k*_*a*_ becomes too large, the survival ratio tends to decrease. This can be related to the fact that for each additional node, one remaining node can be selected multiple times, which reduces the positive effect of node addition.

**Figure 4:**
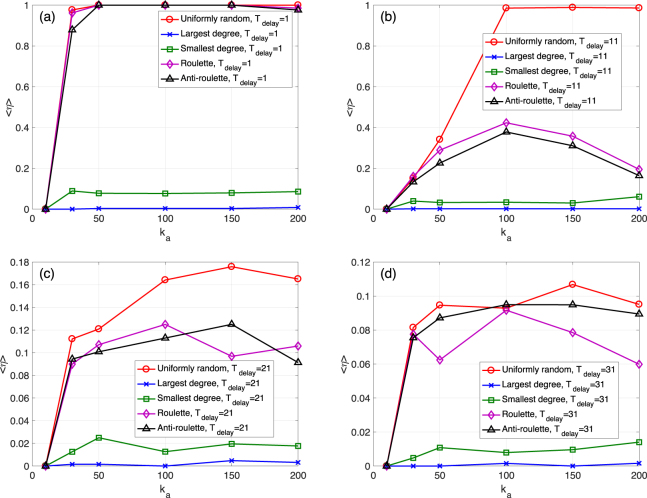
Mean survival ratio 〈*η*〉 as a function of *k*_*a*_ for *N*_*a*_ = 100. (**a**) Different selection rules for the ER network case with *T*_delay_ = 1. *N* = 1000, *M* = 1000, *M*_*a*_ = 10, 〈*k*〉 = 20, *k*_*s*_ = 11, *q* = 0.09, and *f* = 0.1. The threshold for determining a total collapse is *d* = 0.5. (**b–d**) Similar to (a) but for *T*_delay_ = 11, *T*_delay_ = 21, and *T*_delay_ = 31.

Figure 4(a–d) also compare the five node selection rules, but this time we check for different values of *k*_*a*_ as we vary *T*_delay_. For example, Fig. 4(a) shows the results for *T*_delay_ = 1, which is an immediate system recovery, and as we vary *k*_*a*_ between 0 and 200. The uniformly random selection is evidently the best. The roulette/anti-roulette selection has similar but slightly smaller survival ratio values. According to Fig. 4(b–d), for larger *T*_delay_ values, the uniformly random selection is always better than the roulette and anti-roulette selection rules. These results suggest that for restoring an ER network, there is no need to pick nodes to connect to based on degree.

**Figure 5:**
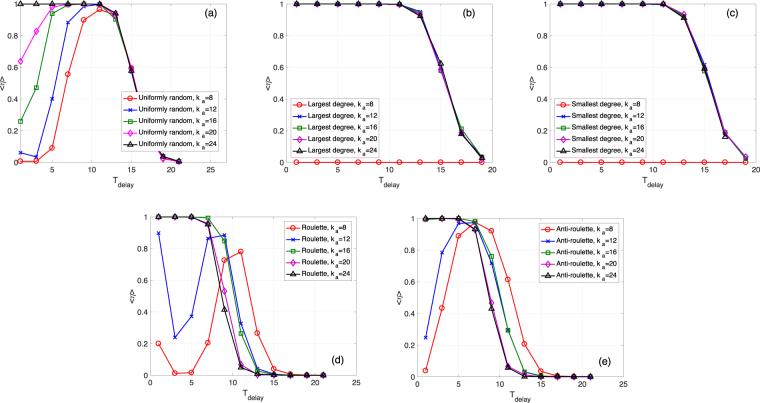
Mean survival ratio 〈*η*〉 as a function of *T*_delay_ for *N*_*a*_ = 100 and different *k*_*a*_ values. (**a**) SF networks with the uniformly random selection. *N* = 1000, *M* = 3000, *M*_*a*_ = 10, *γ* = 1.8, *k*_*s*_ = 5, *q* = 0.39, and *f* = 0.2. The threshold for determining a total collapse is *d* = 0.1. (**b–e**) Similar to (a) but for the largest degree, smallest degree, roulette, and anti-roulette selection rules.

**Figure 6:**
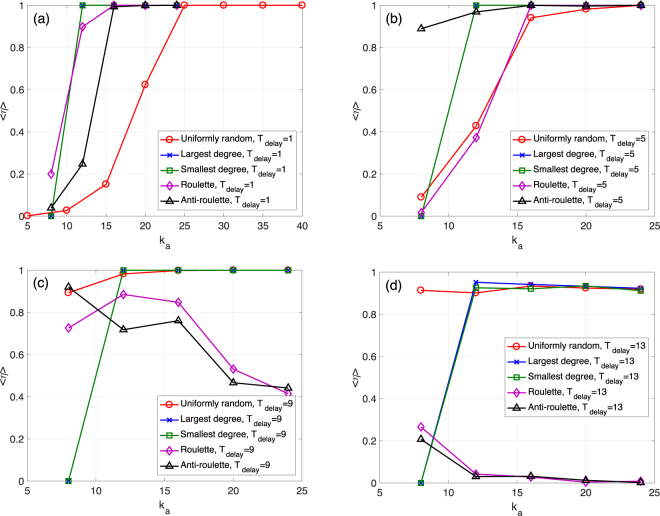
Mean survival ratio 〈*η*〉 as a function of *k*_*a*_ for *N*_*a*_ = 100. (**a**) Different selection rules for the SF network case with *T*_delay_ = 1. *N* = 1000, *M* = 3000, *M*_*a*_ = 10, *γ* = 1.8, *k*_*s*_ = 5, *q* = 0.39, and *f* = 0.2. The threshold for determining a total collapse is *d* = 0.1. (**b–d**) Similar to (a) but for *T*_delay_ = 5, *T*_delay_ = 9, and *T*_delay_ = 13.

In Figs 5 and 6, we present the same as in Figs 3 and 4, but for the SF case. We consider adding *N*_*a*_ = 100 nodes, with different *k*_*a*_ and *T*_delay_ values. In Fig. 5(a), we surprisingly find that for the uniformly random selection, the survival ratio *η* does not monotonically decrease with *T*_delay_, but has a peak at around *T*_delay_ = 11 for different *k*_*a*_ values. This means that to prevent the total collapse of a SF network, sometimes a delayed recovery can be better. As shown in Fig. 5(d,e), the roulette and anti-roulette rules behave similarly. Moreover, we find that, for an immediate node addition, the roulette rule performs better than the other two randomized rules (this will be explained later when we discuss the results in Fig. 6). Finally, as shown in Fig. 5(b,c), the largest degree and smallest degree selection rules perform much better compared to their performance in the ER network case. This is because almost all of the *N*_*a*_ additional nodes and the *k*_*s*_ selected remaining nodes tend to survive when *k*_*a*_ is large enough (compared to *k*_*s*_). Note that *N*_*a*_ + *k*_*s*_ is larger than the threshold *d* = 0.1, which leads to an *η* value of ≈1.

The increasing and decreasing trends of the mean survival ratio in Fig. 5(a) are caused by the fact that increasing *T*_delay_ leads to two competing effects. On the one hand, a larger *T*_delay_ leads to a smaller remaining network before node addition, which tends to cause a smaller final system state after node addition. On the other hand, for larger *T*_delay_, each remaining node on average is connected to more new nodes, which results in larger degree increments for the remaining nodes. To demonstrate this, we show in Supplementary Figs 1 and 2 the distributions of *S*(*t*) and node degrees before adding new nodes, as well as at the final state after node additions, for the ER and SF cases, respectively. Supplementary Fig. 1(a) shows the PDF of *S*(*t*) before node addition for different *T*_delay_ values. Supplementary Fig. 1(b,c) shows the PDF and the CDF of the degree values of the remaining network before adding nodes. Supplementary Fig. 1(d) shows the PDF of the final state after node addition under the uniformly random selection with *N*_*a*_ = 100, *k*_*a*_ = 100 and *M*_*a*_ = 10. Supplementary Fig. 2(a–d) shows the same as Supplementary Fig. 1(a–d) but for the SF network case.

We find that for the ER case, the second trend (larger degree increments) due to increasing *T*_delay_ is weaker. Consequently, for most systems at *T*_delay_ = 21 and *T*_delay_ = 31, the remaining system size, before node addition, plus another 100 nodes remains below the threshold *d* = 0.5. Thus, having larger degree increments does not help increasing the survival ratio in these cases. However, for the SF case, the remaining nodes with small degrees before adding nodes are non-negligible, even for *T*_delay_ = 1. Therefore, having larger degree increments will be more helpful than in the ER case. For *T*_delay_ = 1 and *T*_delay_ = 5, the additional degree to each remaining node is still not large enough for saving them. For *T*_delay_ = 9, thanks the increased degree increments, most final states are not at 0, but around 0.11. This is greater than the threshold *d* = 0.1, which leads to a larger survival ratio *η*. For *T*_delay_ = 13 and *T*_delay_ = 17, the first trend (reduced remaining system size) dominates as in the ER case, consequently most final system states are below the threshold *d* = 0.1.

Similar to Fig. 4, Fig. 6(a–d) compares the the five selection rules for the SF case using different time delay values. For *T*_delay_ = 1, the roulette selection is better than the anti-roulette or the uniformly random one. However, at *T*_delay_ = 5, the anti-roulette is better than the other two randomized rules. When *T*_delay_ becomes larger, the uniformly random selection becomes the best. These results present a different phenomenon compared to the ER case. To interpret these findings, we consider the degree distribution of the surviving network before the node addition is performed for the SF network case. At *T*_delay_ = 1 (see Supplementary Fig. 2(c)), the remaining nodes that fulfil the requirements of being removed are only a small fraction of all remaining nodes. Therefore, it is more important to add links to the original hub nodes to support the connectivity of the remaining network. At *T*_delay_ = 5, the remaining networks before adding nodes include a much larger fraction of nodes with small degrees. Consequently, the anti-roulette rule is better, since it restores more susceptible nodes. Finally, for *T*_delay_ = 9 or *T*_delay_ = 13, the roulette and anti-roulette selection rule are worse than the uniformly random one. This is because both original hub nodes and original nodes with small degrees tend to fulfil the requirements of node removal, These intricate effects of time delay, *T*_delay_, are not observed for the ER network case, since the ER case has homogeneous degree distributions before the node addition.

The above results can be further viewed in light of the total “costs” of the recovery process. Considering that in real world social networks, the cost of introducing one more individual (node) is mainly determined by his/her importance. It costs much more to introduce famous people into the system. Therefore, we can assume that the cost of adding a node is proportional to its degree: number of connections to surviving nodes. This is equivalent to defining the cost of each additional node as *k*_*a*_, and the total costs of the system recovery as *N*_*a*_ ⋅ *k*_*a*_. According to the results presented in Figs 4 and 6, for recovering a homogeneous network, the uniformly random selection rule performs better, since it can reach higher survival ratios at a lower total cost (controlled by the parameter *k*_*a*_). Further, for an early, an intermediate, or a late recovery of a SF network, the roulette, anti-roulette, or the uniformly random selection rules results in larger survival ratios at a lower cost, respectively.

### Tradeoffs between the number of additional nodes and their degree

In this subsection, we investigate the tradeoffs between *N*_*a*_ and *k*_*a*_ for a given fixed total cost value. We can imagine that a larger *N*_*a*_ tends to cause a larger final system state, which is good for system recovery. On the other hand, a larger *k*_*a*_ leads to more robust additional nodes. Therefore, it is important to know which parameter is more critical to the survival ratio *η*. Note that in this subsection we only show the results for the three randomized node selection rules in order to focus on non-trivial results.

**Figure 7:**
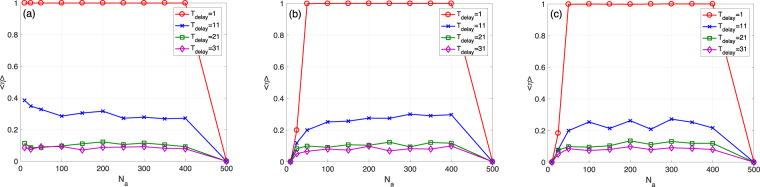
Balance between *N*_*a*_ and *k*_*a*_ for a fixed *N*_*a*_ ⋅ *k*_*a*_. (**a**) Mean survival ratio 〈*η*〉 VS *N*_*a*_ for the uniformly random selection with different *T*_delay_. ER networks. *N* = 1000, *M* = 1000, 〈*k*〉 = 20, *k*_*s*_ = 11, *q* = 0.09, and *f* = 0.1. *N*_*a*_ ⋅ *k*_*a*_ = 5000. The threshold for determining a total collapse is *d* = 0.1. (**b**) Similar to (a) but for the roulette selection. (**c**) Similar to (a) but the anti-roulette selection.

Figure 7(a–c) shows, for the ER case, how the mean survival ratio changes with *N*_*a*_ for a fixed total cost *N*_*a*_ ⋅ *k*_*a*_ = 5000 and a set of *T*_delay_ values. The survival ratio, in the uniformly random selection case, is not strongly affected by *N*_*a*_ for different *T*_delay_, except for a very large *N*_*a*_ (see Fig. 7(a)). This is because, under a fixed total cost, as *N*_*a*_ becomes larger *k*_*a*_ becomes smaller and eventually less than *k*_*s*_ = 11. For the roulette and anti-roulette selection rules, the effect of *N*_*a*_ is similar to the uniformly random selection except for small *N*_*a*_ values.

**Figure 8:**
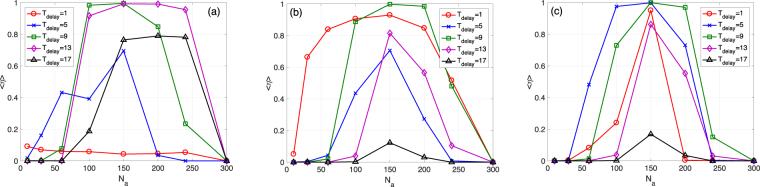
Balance between *N*_*a*_ and *k*_*a*_ for a fixed *N*_*a*_⋅*k*_*a*_. (**a**) Mean survival ratio 〈*η*〉 VS *N*_*a*_ for the uniformly random selection with different *T*_delay_. SF networks. *N* = 1000, *M* = 1000, *γ* = 1.8, *k*_*s*_ = 5, *q* = 0.39, and *f* = 0.2. *N*_*a*_ ⋅ *k*_*a*_ = 1200. The threshold for determining a total collapse is *d* = 0.5. (**b**) Similar to (a) but for the roulette selection. (**c**) Similar to (a) but the anti-roulette selection.

Figure 8(a–c) shows the same as Fig. 7 but for the SF case with a total cost *N*_*a*_ ⋅ *k*_*a*_ = 1200. We find that *N*_*a*_ has a stronger impact on the mean survival ratio 〈*η*〉 than in the ER case. For the uniformly random selection, a very small *N*_*a*_ is preferred at *T*_delay_ = 1. However, the needed number of nodes rises to between 100 and 150 for *T*_delay_ = 5 or *T*_delay_ = 9 and it continues to rise further for *T*_delay_ = 13 and *T*_delay_ = 17 (see Fig. 8(a)). This means that for a more delayed system recovery, a larger *N*_*a*_ and a smaller *k*_*a*_ are needed. In other words, more additional nodes are needed for recovering a system with a smaller remaining size before starting the addition. The roulette and anti-roulette selection rules demonstrate a similar behavior (see Fig. 8(b,c)). These results provide suggestions for restoring near-collapse systems under a fixed total cost.

## Discussion

In this paper, we investigate the possibility of recovering networks that exhibit early warnings of total collapse by adding additional nodes. To this end, we model system collapse using the recently introduced KQ cascade-model and employ the moving standard deviation of the remaining network size time series as an early indicator of an impending cascade. We use five rules for regulating the wiring of the newly added nodes to existing nodes. These include three random rules: uniformly random, roulette and anti-roulette. The latter two connect a new node to a set of randomly selected existing nodes with a probability proportional and inversely proportional, respectively, to their degree in the original network. The five rules include also two deterministic rules that connect new nodes to existing nodes with largest and smallest degrees in the original network, respectively. We find that an early addition of nodes (i.e. immediately after observing early warning signals) is always better for preventing ER networks from a total collapse. This is because ER networks are characterized by a homogeneous degree distribution. SF networks, however, benefit more from a delayed intervention, that is to start adding nodes after a certain time delay *T*_delay_. Investigating the interplay between the five connection rules and *T*_delay_ shows that the uniformly random selection is always the best strategy for saving ER networks. For SF network, the best wiring rules change from roulette to anti-roulette, and finally to the uniformly random rule as *T*_delay_ increases. This complex interplay is a product of node degree heterogeneity in SF networks. Finally, we explore the balance between the number of needed nodes *N*_*a*_ and their degree *k*_*a*_ that are needed for restoring a collapsing system at a fixed cost of *N*_*a*_ ⋅ *k*_*a*_. We find that SF networks need to add more nodes as *T*_delay_ increases. However, *N*_*a*_ has minimal impact on ER networks survival.

Our findings provide insights into saving networks that are predicted to approaching a total collapse. For example, the counterintuitive results of SF networks restoration, i.e. the positive impact of time delay, can be applied to social structures (companies) and networks with impending cascade to prevent a total collapse. Note that many real-world social networks are known to have heterogeneous structures.

Going forward, we plan to apply the proposed network recovery framework to other sorts of cascading failure models. These include overload based cascades^10,20^, which are known to exhibit a slow down near criticality. Furthermore, while the KQ-cascade and node addition based-recovery are more related to social networks like Facebook, it will be interesting to investigate failure models and recovery scenarios that are relevant to other systems. For example, cascades based on dependencies or overloads, with recovery by reconnecting failed nodes^29,30,32,33^, are more applicable to systems with physical connections, such as the power-grid and traffic systems.

## Electronic supplementary material

Supplementary Information

## References

[1] Buldyrev SV, Parshani R, Paul G, Stanley HE, Havlin S (2010). Catastrophic cascade of failures in interdependent networks. Nature.

[2] Parshani R, Buldyrev SV, Havlin S (2010). Interdependent networks: reducing the coupling strength leads to a change from a first to second order percolation transition. Physical Review Letters.

[3] Parshani R, Buldyrev SV, Havlin S (2011). Critical effect of dependency groups on the function of networks. Proc. Natl. Acad. Sci. USA.

[4] Gao J, Buldyrev SV, Stanley HE, Havlin S (2012). Networks formed from interdependent networks. Nature Physics.

[5] Hu Y, Ksherim B, Cohen R, Havlin S (2011). Percolation in interdependent and interconnected networks: Abrupt change from second- to first-order transitions. Physical Review E.

[6] Hu Y (2013). Percolation of interdependent networks with intersimilarity. Physical Review E.

[7] Reis SD (2014). Avoiding catastrophic failure in correlated networks of networks. Nature Physics.

[8] Feng L, Monterola CP, Hu Y (2015). The simplified self-consistent probabilities method for percolation and its application to interdependent networks. New Journal of Physics.

[9] Yuan X, Hu Y, Stanley HE, Havlin S (2017). Eradicating catastrophic collapse in interdependent networks via reinforced nodes. Proceedings of the National Academy of Sciences.

[10] Motter AE, Lai Y-C (2002). Cascade-based attacks on complex networks. Physical Review E.

[11] Crucitti P, Latora V, Marchiori M (2004). Model for cascading failures in complex networks. Physical Review E.

[12] Motter AE (2004). Cascade control and defense in complex networks. Physical Review Letters.

[13] De Martino D, Dall’Asta L, Bianconi G, Marsili M (2009). Congestion phenomena on complex networks. Physical Review E.

[14] Brummitt CD, D’Souza RM, Leicht EA (2012). Suppressing cascades of load in interdependent networks. Proceedings of the National Academy of Sciences.

[15] Tan F, Xia Y, Zhang W, Jin X (2013). Cascading failures of loads in interconnected networks under intentional attack. Europhysics Letters.

[16] Li D, Jiang Y, Kang R, Havlin S (2014). Spatial correlation analysis of cascading failures: congestions and blackouts. Sci. Rep..

[17] Tan F, Wu J, Xia Y, Tse CK (2014). Traffic congestion in interconnected complex networks. Physical Review E.

[18] Chen Z, Zhang J, Du W-B, Lordan O, Tang J (2015). Optimal allocation of node capacity in cascade-robustness networks. PLoS ONE.

[19] Xia Y, Zhang W, Zhang X (2016). The effect of capacity redundancy disparity on the robustness of interconnected networks. Physica A.

[20] Zhao J, Li D, Sanhedrai H, Cohen R, Havlin S (2016). Spatio-temporal propagation of cascading overload failures in spatially embedded networks. Nature Communications.

[21] Zhou D, Elmokashfi A (2017). Overload-based cascades on multiplex networks and effects of inter-similarity. PLoS ONE.

[22] Garcia, D., Mavrodiev, P. & Schweitzer, F. Social resilience in online communities: The autopsy of friendster. In *Proceedings of the first ACM conference on Online social networks*, 39–50 (ACM, 2013).

[23] Dorogovtsev SN, Goltsev AV, Mendes JFF (2006). K-core organization of complex networks. Physical Review Letters.

[24] Bak P, Tang C, Wiesenfeld K (1988). Self-organized criticality. Physical Review A.

[25] Goh K-I, Lee D-S, Kahng B, Kim D (2003). Sandpile on scale-free networks. Physical Review Letters.

[26] Lee K-M, Goh K-I, Kim I-M (2012). Sandpiles on multiplex networks. Journal of the Korean Physical Society.

[27] Noël P-A, Brummitt CD, D’Souza RM (2013). Controlling self-organizing dynamics on networks using models that self-organize. Physical Review Letters.

[28] Majdandzic A (2014). Spontaneous recovery in dynamical networks. Nat. Phys..

[29] Liu C, Li D, Zio E, Kang R (2014). A modeling framework for system restoration from cascading failures. PLoS ONE.

[30] Liu C (2014). Modeling of self-healing against cascading overload failures in complex networks. Europhysics Letters.

[31] Böttcher L, Lukovic′ M, Nagler J, Havlin S, Herrmann H (2017). Failure and recovery in dynamical networks. Sci. Rep..

[32] Di Muro MA, La Rocca CE, Stanley HE, Havlin S, Braunstein LA (2016). Recovery of Interdependent Networks. Sci. Rep..

[33] Majdandzic A (2016). Multiple tipping points and optimal repairing in interacting networks. Nature Communications.

[34] Zhou D (2014). Simultaneous first-and second-order percolation transitions in interdependent networks. Physical Review E.

[35] Baxter GJ, Dorogovtsev SN, Lee K-E, Mendes JFF, Goltsev AV (2015). Critical dynamics of the *k*-core pruning process. Phys. Rev. X.

[36] Lee D, Choi W, Kertész J, Kahng B (2017). Universal mechanism for hybrid percolation transitions. Sci. Rep..

[37] Dakos V (2008). Slowing down as an early warning signal for abrupt climate change. Proceedings of the National Academy of Sciences.

[38] Scheffer M (2009). Early-warning signals for critical transitions. Nature.

[39] Dai L, Vorselen D, Korolev KS, Gore J (2012). Generic indicators for loss of resilience before a tipping point leading to population collapse. Science.

[40] Scheffer M (2012). Anticipating critical transitions. Science.

[41] Dakos V, Bascompte J (2014). Critical slowing down as early warning for the onset of collapse in mutualistic communities. Proceedings of the National Academy of Sciences.

[42] van de Leemput IA (2014). Critical slowing down as early warning for the onset and termination of depression. Proceedings of the National Academy of Sciences.

[43] Podobnik B (2015). Predicting the Lifetime of Dynamic Networks Experiencing Persistent Random Attacks. Sci. Rep..

[44] Yu Y (2016). System crash as dynamics of complex networks. Proceedings of the National Academy of Sciences.

