# Supplementary material for: Network recovery based on system crash early warning in a cascading failure model
Source: Sci Rep. 2018 May 10;8:7443. doi: 10.1038/s41598-018-25591-6 (PMC5945858; doi:10.1038/s41598-018-25591-6)
Supplement: Supplementary file 1 — Supplementary Information [file 41598_2018_25591_MOESM1_ESM.pdf]

Supplementary Information

Network recovery based on system crash early warning in a cascading failure model

Dong Zhou and Ahmed Elmokashfi

Supplementary Figures

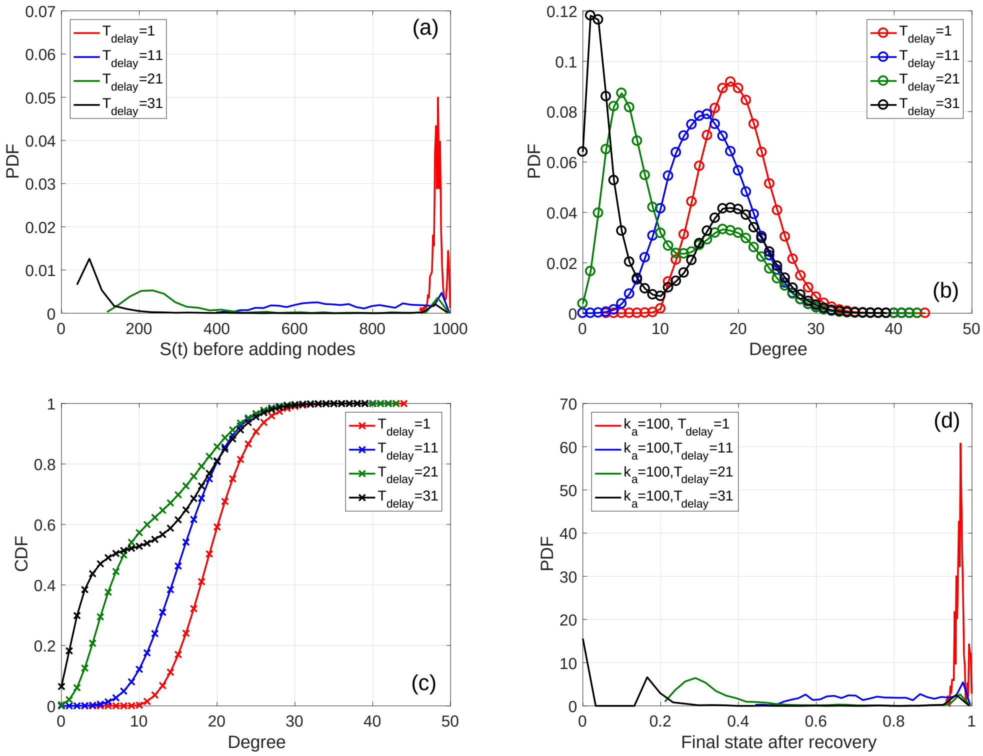

Supplementary Figure S1: Distributions of the remaining network sizes and degree values. **a**. PDF of $S(t)$ at $t = T_{\text{pred}} + T_{\text{delay}}$ before the node addition. ER networks. $N = 1000$, $M = 1000$, $\langle k \rangle = 20$, $k_s = 11$, $q = 0.09$, and $f = 0.1$. The threshold for determining a total collapse is $d = 0.5$. **b**. The same with **a**. but for the degree values of the surviving nodes before the node addition. **c**. The same with **b**. but the CDF. **d**. PDF of $S(t)$ at the end of the cascading failure process after node addition with the uniformly random selection. $N_a = 100$, $M_a = 10$, and $k_a = 100$ are considered here.

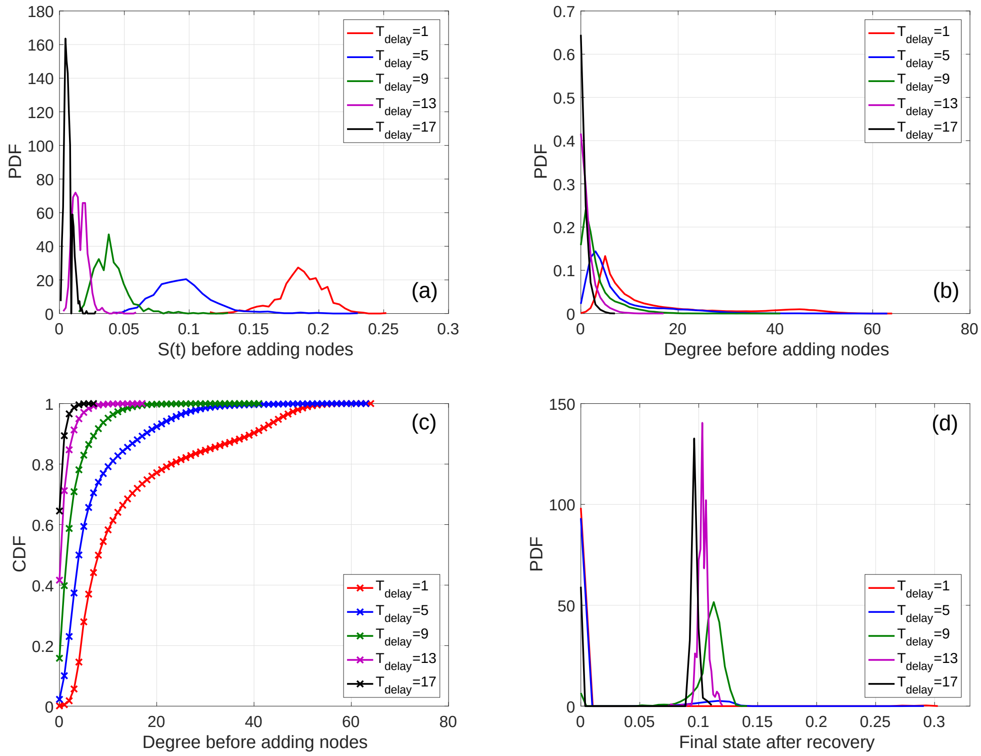

Supplementary Figure S2: Distributions of the remaining network sizes and degree values. **a**. PDF of $S(t)$ at $t = T_{\text{pred}} + T_{\text{delay}}$ before the node addition. SF networks. $N = 1000$, $M = 1000$, $\gamma = 1.8$, $k_s = 5$, $q = 0.39$, and $f = 0.2$. The threshold for determining a total collapse is $d = 0.1$. **b**. The same with **a**. but for the degree values of the surviving nodes before the node addition. **c**. The same with **b**. but the CDF. **d**. PDF of $S(t)$ at the end of the cascading failure process after node addition with the uniformly random selection. $N_a = 100$, $M_a = 10$, and $k_a = 100$ are considered here.
